# Speech Features and Electroencephalogram Parameters in 4- to 11-Year-Old Children

**DOI:** 10.3389/fnbeh.2020.00030

**Published:** 2020-03-16

**Authors:** Elena Lyakso, Olga Frolova, Yuri Matveev

**Affiliations:** Laboratory of Child Speech Research Group, Department of Higher Nervous Activity and Psychophysiology, Saint Petersburg State University, Saint Petersburg, Russia

**Keywords:** electroencephalogram, brain activity, acoustic features, linguistic characteristics, child speech

## Abstract

The goal of the study is to investigate a correlation between different levels of speech organization, indicating the physiological processes of maturation of the vocal tract structures and brain regions associated with speech and language, and basic electroencephalogram (EEG) rhythms, reflecting the age-related dynamics of maturation of brain structures in children aged 4–11 years. The complex method of analysis, including EEG registration, clinical and spectral analysis of EEG; dichotic listening, identifying the profile of functional lateral asymmetry (PFLA), and phonemic hearing of the child; recording, linguistic, and acoustic analysis of child speech; and identification of speech characteristics reflecting the formation of its different levels, was used. Two complementary experimental series were conducted: the correlation between EEG parameters, speech features, dichotic listening, the PFLA, and phonemic hearing of the child in the age dynamics of 4–11 years (first); the specificity of EEG patterns in children at different stages of reading skills formation (second). The result of this study showed the correlation between acoustic and linguistic features of child speech and brain activity. The analysis of EEG and acoustic features of child speech revealed the correlation between pitch and pitch range values in spontaneous speech and theta-rhythm intensity in EEG. High values of pitch and its variation in younger children (4–6 years) are related to the intensity of theta rhythm in the EEG pattern, as this rhythm is most expressed in younger children. It was revealed that the alpha rhythm is asymmetrically localized in children with clear pronunciation of words (which determines the intelligibility of their speech) that is typical for 6.5- to 11-year-old children. The formation of reading skills in a child is associated with a change in the characteristics of the alpha rhythm—from irregular, unstable, low frequency, and low amplitude in children at the beginning of reading skills mastering to medium and low amplitude, regular, asymmetrically localized in children reading words and phrases. The specifics of the relation between brain activity and different levels of speech formation at different child’s age periods are discussed.

## Introduction

The process of normative development of a child is associated with anatomical and physiological changes in various organs and structures that have the specificity in the age dynamics, and with social and environmental factors that determine the realization of the biological basis.

A physiological indicator characterizing the child’s willingness for information perception and processing is the maturation of brain structures and, as a result, the formation in the resting-state electroencephalogram (EEG) of the regular alpha as a dominant form of brain activity (Farber and Njiokiktjien, 1993; Farber et al., 2000).

Within 3 months after birth in newborns, rhythmic activity is gradually forming. Delta waves prevail in the EEG (Eisermann et al., 2013). Delta amplitude decreases with child’s age. The irregular diffuse activity of medium voltage, which is present in the neonatal period, is gradually replaced by more rhythmic theta waves to the end of the first year of life (Eisermann et al., 2013). Slow rhythms are replaced by faster alpha rhythm between 12 months old and 7–8 years old. Theta decreases and alpha increases as a function of age (Clarke et al., 2001). In waking-state EEG in 1–3 year olds, the posterior basic rhythm increases from theta frequencies to lower alpha range with high individual variability (Eisermann et al., 2013). The theta band dominates in the EEG pattern of 3- to 4-year-old children (Perone et al., 2018b). Between age of 7 and 10 years, theta is dominant in posterior areas (Puligheddu et al., 2005). Intermixed posterior slow activity is still present at the age of 6 years and clearly diminishes after the age of 12 years (Eisermann et al., 2013). For the delta, theta, and alpha rhythms, maturation begins in posterior regions and ends in anterior regions (Barriga-Paulino et al., 2011; Perone et al., 2018b).

Alpha peak frequency increases over the posterior regions from about 4 to 8 Hz from infancy to early childhood (Marshall et al., 2002) and from about 9 to 10 Hz from middle childhood to early adolescence (Miskovic et al., 2015). At the age of 10 years, alpha rhythm reaches 10 Hz, which is equal to the mean frequency of the adult EEG (Niedermeyer, 2005), and is located in frontal, central, and occipital areas.

For 6- to 7-year-old children, organized parieto-occipital alpha, which reflects the maturation of corresponding brain structures, is one of the indicators of the child’s readiness for learning and implementing cognitive tasks (Farber et al., 2000; Bezrukikh et al., 2009). Alpha rhythm is dominant after the age of 8 years. It is present over all areas in the EEG of 7- to 9-year-old children, but its largest values are recorded in the occipital areas (Puligheddu et al., 2005).

The alpha amplitude increases and alpha power decreases with children’s age (Marshall et al., 2002; Miskovic et al., 2015). An age-related decrease in absolute EEG power is shown, which coincides with a decrease in the volume of gray matter (Whitford et al., 2007). It is revealed that maturational changes in structure and neurotransmitter function occur in the brain of adolescents (Segalowitz et al., 2010). Alpha rhythm estimation in 9- to 23-year-old participants showed that alpha power decreased from the age of 9–12 years to 13–17 years. It was found that the later pubertal stage is associated with a decrease in alpha power in the prefrontal and occipital areas in men but not in women (Howsley and Levita, 2018), which is confirmed by the evidence that the maturation of the alpha rhythm is not completed until 16 years (Marcuse et al., 2008).

Thus, the main changes in brain maturation with the age of the child are transition from delta and theta, specific for children, to alpha and beta, specific for adults and decrease in the absolute EEG power.

Many studies have focused on the connection between rhythm and cognitive development (Perone et al., 2018a), mainly alpha and gamma (Bell, 2001; Benasich et al., 2008; Gou et al., 2011; Cuevas et al., 2012). Individual differences in alpha power over the frontal area are associated with cognitive development (Bell and Fox, 1992). Children of 4 years of age, who had a high level of alpha during the assignment compared to the previous base period of rest, showed higher abilities to perform cognitive functions (Cuevas et al., 2012). It was indicated that the peak frequency in the alpha range is associated with premature reading ability (Suldo et al., 2002).

Cognitive development level is most clearly reflected in child’s speech skills mastering. The speech development in ontogenesis is due, on the one hand, to the maturation of brain structures (Amunts et al., 2003), the development of the perception system (Eisenberg et al., 2000; Moore, 2002; Bishop et al., 2011), the growth of the vocal tract structure (Martland et al., 1996; Robb et al., 1997; Vorperian and Kent, 2007) and, on the other hand, to social factors and culture.

From birth, the vocal tract structures grow, the conformation of its parts changes (Kent and Murray, 1982; Smith and Goffman, 2004; Vorperian and Kent, 2007), and the motor control of articulatory and respiratory movements is installed, which leads to developing the system of vowels and consonants and their articulation accuracy in words. Phonetic mastering is considered to be completed at the age of 8 years, but the speech development in its subtle aspects, including acoustic, prolongs until late adolescence (Smith and Goffman, 2004; Vorperian and Kent, 2007). The formation of articulation skills is reflected in the acoustic features of a child’s speech—values of formants, the vowel articulation index (VAI) (Lyakso et al., 2016, 2017; Lyakso and Frolova, 2018). On the material of Russian language, it was shown that during the third year of life, the vowel stress is formed. In all the vowels, the stressed vowel’s duration tends to be longer than that of the unstressed one. The palatalized versus non-palatalized consonant opposition development begins, which is found in the characteristics of vowels following them (Lyakso et al., 2003). Despite the still high pitch values, they differ from stressed to unstressed vowels at 4 years of age (Lyakso and Gromova, 2005). The length of the stressed vowel is significantly higher compared to the unstressed vowels in words of 6 and 7 year olds. By the age of seven, a stressed vowel in words is distinguished by its length meaning, as a relevant feature for the Russian language (Lyakso et al., 2009). Changes in the size and configuration of the vocal tract, which are associated with age-related changes in the growth of the child, and improvement in the motor control lead to a decrease in the pitch values and pitch variation (Lyakso and Grigorev, 2015).

The maturation of various brain regions occurs unevenly—the visual cortex gets matured by age of 7 years, the middle frontal gyrus does not reach the adult level up to 17 years, the auditory cortex reaches maturity by age of 12 years (Huttenlocher, 1990; Huttenlocher and Dabholkar, 1997), and speech centers of the temporal cortex continue to develop during adolescence (Gogtay et al., 2004). The brain is up to 95% of the size and weight of an adult brain at the age of 7–11 years (Caviness et al., 1996; Giedd et al., 1996). It was shown that the gyrification index (the ratio of the number of gyri to the total surface of the brain) reaches adult values during the second decade of life (Armstrong et al., 1995). Fractional anisotropy showed that, with age, the quantity of connections increases in the main paths that provides functional integration, fine motor control, and speech information processing (Schmithorst et al., 2002; Ashtari et al., 2007). All these processes are the physiological basis for the cognitive and speech development of the child. The age from 4 to 8 years is characterized by the complication of grammar, syntax, and pragmatics of speech of children. Understanding the structure and shape of a spoken language is formed (Adams, 1990), and speech processing skills are developed, providing the basis for mastering literacy (Parviainen et al., 2011) and reading skills (Lyakso et al., 2010).

Thus, as follows from the presented review, maturation of brain activity, cognitive development, and different aspects of speech development of the child are considered as separate studies. Given the fact that the central nervous system (CNS) maturation rates and vocal tract age changes have a wide variation even in the normal development of children, it is important to identify a correlation between child’s age, EEG characteristics (alpha, theta, and delta rhythms, most clearly reflecting age-related changes in brain activity), and the features of child’s speech development.

The goal of our study is to investigate a correlation between different levels of speech organization, indicating the physiological processes of maturation of the vocal tract structures and brain regions associated with speech and language, and basic EEG rhythms, reflecting the age-related dynamics of maturation of brain structures in children aged 4–11 years.

The main tasks of the study are as follows:

1. to characterize the speech development of children in age dynamics;
2. to describe the age dynamics of the EEG pattern by the characteristics of the main rhythms: alpha, theta, and delta;
3. to identify the relationship between the characteristics of EEG rhythms, speech features and dichotic listening, the profile of functional lateral asymmetry (PFLA), and phonemic hearing associated with speech in the age dynamics of 4–11 years;
4. to describe the features of brain activity—on the basis of alpha rhythm and the formation of reading skills in children aged 4–7 years.

## Materials and Methods

### Participants of the Study

In the study, 64 children aged 4–11 years participated. Two studies were conducted.

Study 1: “The correlation between EEG parameters, speech features, and dichotic listening, identifying the PFLA, phonemic hearing of the child”—64 children aged 4–11 years (44 boys and 20 girls) were the participants. According to pediatricians’ conclusions, the children developed normally: gestational age—38 ± 1.5, 36–41 weeks, the Apgar score of 8–9 (7.5 ± 0.9–8.5 ± 0.8); birth weight—3,192 ± 556.7 g, 2,100–4,400 g. The children were divided into three groups in accordance with their age: group 1, 4–6 years old (preschool age)—4.9 ± 0.87 years (*n* = 35 children); group 2, 6.5–8 years old (the first to second grades in the elementary school)–7.0 ± 0.6 years (*n* = 17 children) (in Russia, school begins at child’s age of 6.5 years); and group 3, 9–11 years old (the third to fifth grades in the school)–10.6 ± 0.94 years (*n* = 12 children). The choice of age range is due to the main stages of child development, i.e., preschool, elementary school, middle school (up to active puberty), which are characterized by anatomical, physiological, functional features, cognitive development, and social relationships. According to the physiological criteria, i.e., the Apgar score, height, and weight, children included in different groups do not significantly vary.

Study 2: “The peculiarities of EEG pattern in children at different stages of reading skills formation”—the participants were 19 children aged 4–7 years from Study 1.

### The Design of the Study

The design of Study 1 is as follows: audio and video recording of the spontaneous speech of the child, the dialogue of the experimenter with the child, and reading; dichotic listening, the PFLA, and phonemic hearing of the child; and EEG recording.

The design of Study 2 is as follows: the testing for the child’s ability to read the text of varying difficulty (letters, syllables, words, and phrases) using the ABC book and the fairy tale “Little Red Riding Hood”; examination the formation of the child’s attention on the meaning of the text and understanding words and phrases by selecting pictures to the read text; and EEG recording. In Study 2, registration of physiological indicators and reading process were separated in time.

### Methods

The classical analysis methods were used in the work: clinical and spectral analysis of EEG and analysis of different levels of child’s speech formation. The original approach and the novelty of the study are caused by the combination of classical methods of EEG and speech analysis in one work to reveal the correlation between obtained parameters in the age dynamics of 4–11 years.

#### EEG Recording and Analysis

The complex method of analysis includes registration and analysis of EEG, dichotic listening, identifying the PFLA, phonemic hearing of the child, and the speech features, and reflecting the formation of its different levels. All studies were conducted in the same conditions in the laboratory.

Electroencephalogram recording was carried out using the standard visual load. Preparing the children to the study was different depending on the child’s age, but it was identical for all children of the same age.

Two experimental studies are related to participating children in studies with different cognitive tasks: (1) the correlation between EEG parameters, speech features, dichotic listening, the PFLA, and phonemic hearing of the child in the age dynamics of 4–11 years; and (2) the peculiarities of EEG pattern in children aged 4–7 years at different stages of reading skills formation.

Electroencephalogram recording was standardized for all participants of the study. The same design of EEG study was used. Dichotic listening, identifying the PFLA, phonemic hearing, and the dialogue with the adult to assess the child’s speech development and reading to assess reading skills (for some children) were conducted before EEG recording.

##### EEG recording

Resting-state EEG recording of children was carried out using the electroencephalographs “Mitsar-EEG-201” and “Mitsar-EEG-202.” The frequency band was used in the range of 0.53–30 Hz with the amplifier sensitivity of 50 μV/cm. The International 10–20 system of electrode placement was applied. Active electrodes were installed in accordance with the system in leads: Fp1, Fp2, F3, F4, Fz, F7, F8, C3, C4, Cz, T3, T4, P3, P4, Pz, T5, T6, O1, and O2. Reference electrodes were attached to earlobes (A1 and A2); the ground electrode was centered on the forehead. The active electrodes were standard silver–chloride electrodes. After checking the EEG record quality, we proceeded to its computer recording, which was carried out in a calm state with eyes closed in several stages.

Electroencephalogram recording included the following: The background EEG is recorded for 1–3 min, after which the EEG is recorded using the functional load—eye closure/opening, 1 min background recording, photostimulation at frequencies from 1 to 20 Hz (to determine the frequency band where rhythm assimilation is possible), 1 min background recordings, 3 min of hyperventilation load, and 1 min of background recording. Hyperventilation was used as a provoking factor to detect changes of a borderline or pathological in the CNS. Hyperventilation were used sparingly: the child’s mouth had to be open, the breathing rate was no more than 20 cycles per minute, and the depth of breathing had to be uniform, without pants. The child had to sit with his eyes closed. During hyperventilation, exercises were stopped at the appearance of paroxysmal-like or slow high-amplitude activity in the EEG pattern.

Before starting EEG recording, during the fixation of electrode caps and electrodes on the child’s head, children aged 4–6 years watched a cartoon. EEG in younger children was recorded in the presence of one of their parents. It is difficult for small children to sit long with their eyes closed. Therefore, for EEG registration, we used a game in which children prepare to fly into outer space on a rocket ship (eyes closed) and then look at outer space (eyes open). A similar design is used in another study (Perone et al., 2018b). Children 7–11 years old were not in need of the game situation during EEG recording and watching a cartoon during EEG preparing.

The clinical and spectral analysis of EEG was made. EEG analysis was conducted on the basis of the “EEG-2000” software version 3.0.

##### Clinical analysis of EEG

We focused on describing the characteristics of alpha, theta, and delta rhythms. Three main rhythms were selected: alpha, theta, and delta as rhythms reflecting the maturation of brain structures and cortico-subcortical connections. In adults, the alpha rhythm oscillation frequency varies from 8 to 13 Hz; its amplitude is 5–100 μV. The alpha is mainly recorded in the occipital and parietal areas of the cerebral cortex. The delta rhythm (slow waves)—the oscillation frequency from 1 to 4 Hz, amplitude of 20–200 μV (high-amplitude waves)—is associated with a low level of activation. The theta rhythm—the oscillation frequency from 4 to 8 Hz, amplitude of 20–100 μV—is registered in the frontal areas and the hippocampus (Niedermeyer, 2005). The main focus in the work is on alpha-rhythm analysis because this rhythm represents the brain maturity; its stability shows the readiness to information perception. Characteristics of rhythms were evaluated according to the degree of expression, alpha asymmetry, and localization.

Background activity was analyzed (with eyes closed). The visual EEG analysis was performed by three experts using the “EEG-2000” software version 3.0. During the visual analysis, the expression, regularity, modulation, and spatial distribution of the alpha rhythm were determined. The presence and characteristics of slow- and high-frequency rhythms were revealed.

##### Spectral analysis of EEG

For the spectral analysis, EEG record fragments with an average length of 6 s were selected; the epoch was 2 s without overlapping the Hann window. The alpha peak frequency was defined based on the special program using the “EEG-2000” software version 3.0.

For the statistical analysis, relative values based on data processing were used: rhythm intensity—for alpha, 1 = unexpressed, 2 = irregular, unstable, 3 = regular, unstable, and 4 = regular, stable; for theta and delta, 1 = expressed, 2 = irregular; for alpha asymmetry, 1 = left-sided and 2 = right-sided, central (no localization); for localization, 1 = generalized, 2 = mainly in the parieto-occipital area, and 3 = mainly in the centro-parietal area.

Criteria used for EEG parameters ranking were the following:

Rhythm intensity for alpha—1 = unexpressed or weak irregular alpha-like activity with an unclear focus in occipital and/or parietal areas, frequency of 8.3–10.5 Hz, and amplitude of 60–120 μV; 2 = irregular unstable; focus in occipital, parietal, parieto-temporal, parieto-occipital, and centro-parietal, frequency of 8–10.7 Hz, amplitude of 50–120 μV, index of 20–70%; 3 = regular unstable, focus in occipital, parietal, and parieto-occipital areas, frequency of 8.3–10.7 Hz, amplitude of 70–120 μV, index of 60–90%; 4 = regular stable, parieto-occipital, occipital, frequency of 8.3–11.5 Hz, amplitude of 60–100 μV, and index of 60–100%;

Rhythm intensity for theta—1 = expressed, frequency of 4–7.8 Hz, amplitude of 40–100 μV (generalized, parieto-occipital, parietal, parieto-temporal, centro-parietal); 2 = irregular, frequency of 4–7 Hz, amplitude of 40–70 μV (generalized, generalized with bursts in antero-central zones, focus in fronto-central areas, both antero-central and postero-central areas, mainly in antero-central areas);

Rhythm intensity for delta—1 = expressed, frequency of 1.8–4 Hz, amplitude of 50–200 μV; 2 = irregular, frequency of 2–4 Hz, amplitude 30–100 μV;

For all slow-wave rhythms (in total)—1 = index of 50–95%, variants were theta-rhythm dominates (100 μV), irregular activity is marked in the delta range, delta activity prevails (50 μV), and theta-rhythm bursts are observed (up to 100 μV); 2 = index of 15–95%.

#### Lateral Asymmetry and Phonemic Hearing Methods

The leading hemisphere by speech and the PFLA were determined; phonemic hearing was tested as methods associated with speech development.

Dichotic listening is a psychological test commonly used to investigate selective attention and the lateralization of brain function within the auditory system. It is used as a behavioral test for hemispheric lateralization of speech perception. Through the stereo headphones “HD 415” in the “Cool Edit Pro” software, some words were presented in one ear; simultaneously, other words were in the other ear. The child was required to repeat the word (words) that he/she heard out loud; it was recorded on the “Marantz PMD660.” Sixty pairs of words combined into a sequence with a 3-s interval between pairs were used as stimuli. The coefficient of lateral preference (CLP) was calculated using the formula:

$$\text{CLP}=\frac{\left(R\,-\,L\right)\,\times \,100}{\left(R+\,L\right)}$$

(in%), where *R* is the number of “right choices” (words uttered by the child from the stimuli given to the right ear), and *L* is the number of “left choices” (words uttered by the child from the stimuli given to the left ear). If the CLP values were in the range of −10 to +10%, no preference–ambivalence was found; less than −10%, the left-sided preference (the dominance of the right hemisphere); more than +10%, the right-sided preference (the dominance of the left hemisphere).

The PFLA was determined according to tests that reveal the use of the leading arm, leg, eye, and ear. A standard set of tasks was used. For each child, the asymmetry coefficient for each task and the total coefficient using the CLP formula (presented in the description of dichotic listening) were calculated.

Evaluating phonemic hearing was carried out using the software based on pairs and triples of syllables used in speech therapy.

#### Speech Recording and Analysis

To assess the level of speech development of the child, the recording and analysis of child’s speech were carried out. For child speech recording, child speech was recorded by the digital recorder “Marantz PMD660” with external microphone “SENNHEIZER e835S” and video camera “SONY HDR-CX560E.” Speech files were stored in Windows PCM format WAV, 44.100 Hz, 16 bits per sample; video files were in AVI format. The recording was carried out in situations of spontaneous speech, dialogues with adults on given topics, and reading.

Depending on the child’s age, the duration of child’s speech recording was 20–40 min. The spontaneous speech included the monolog of a child on topics about favorite activities, games, and friends. The dialogue between the experimenter and child included the standard set of questions addressed to the child. The experimenter began the dialogue with the request to say the child’s name and age. Then, the experimenter consistently asked questions:

1. Do you like to go to school/kindergarten?
2. What do you like in school/kindergarten (classes or play with friends)?
3. What are your favorite tasks? Why?
4. Do you have any hobbies?
5. What are your favorite movies, cartoons, books, games?
6. Do you have brothers or sisters?
7. Do you have pets?
8. Did you visit the zoo, circus, and museum?

All the children read the fragment of the fairy tale, “Little Red Riding Hood.”

The spectrographic analysis of speech and the text transcription were carried out. The syntactic structure of the response replicas was described as one word, simple phrase, two phrases, complex sentence, and several phrases. The frequency of different types of response replicas was calculated.

Spectrographic analysis of speech was made in “Cool Edit Pro” sound editor. The spectrographic analysis includes the following: the duration of words, stressed and unstressed vowels in words, values of pitch (F0) (average, maximal, minimal for vowels and average values for the stationary part of vowels) were measured. The pitch range (F0maximum−F0minimum) of the vowel and first two formants values (F1, first formant; F2, second formant) at the stationary part of vowels were measured. The temporal and spectral characteristics of speech were automatically calculated, based on the algorithms implemented in the Cool Edit Pro sound editor. These acoustic features reflect the main physiological processes in the vocal tract during voice and speech formation. The temporal characteristics reflect the processes of formation of speech breathing, the pitch values—the frequency of oscillations of vocal folds, the values of the first two formants—the articulation movements in the oral cavity. The values of the first two formants of vowels are acoustic keys to identify vowels.

The accuracy of vowel articulation was determined on the basis of values of VAI (Roy et al., 2009):

$$\text{VAI}=\frac{\text{F}\,1\,\left[\text{a}\right]+\text{F}\,2\,\left[\text{i}\right]}{\text{F}\,1\,\left[\text{i}\right]+\text{F}\,1\,\left[\text{u}\right]+\text{F}\,2\,\left[\text{a}\right]+\text{F}\,2\,\left[\text{u}\right]}$$

where F1[*x*] and F2[*x*] are the values of the first and the second formants of the corresponding vowels, respectively.

#### Statistic Data Analysis

Statistical analysis were performed using “STATISTICA-10.” We used non-parametric criteria: Spearmen correlation (*p* < 0.05), Mann–Whitney test, regression analysis, and multiple regression analysis. The aim of the use of statistical analysis methods is to reveal correlations between indicators of the child’s speech development and EEG characteristics in the age aspect. For this purpose, Spearman correlation analysis was used. The regression analysis was used to confirm the correlations revealed by Spearman. Multiple regression analysis was conducted (1) to rank the contribution of speech development indicators (independent or predictor variables) to a characteristic of a certain age (dependent variable) and (2) to determine the relationship between EEG characteristics (independent variables) and indicators of the child’s speech development (dependent variable).

All procedures were approved by the Health and Human Research Ethics Committee (HHS, IRB 00003875, Saint Petersburg State University), and written informed consent was obtained from parents of the child participant.

## Results

### Study 1—The Correlation Between EEG Parameters, Speech Features, Dichotic Listening, the PFLA, Phonemic Hearing of the Child in the Age Dynamics of 4–11 Years

Analysis of the voice and speech characteristics of children showed a decrease in the pitch values with the age of children (Table 1). The VAI for stressed vowels in words has the highest value in children of the middle age group (1.02) and the smallest values in children aged 9–11 years (0.93). VAI correlates with pitch values (−0.79, Spearman correlation *p* < 0.05)—the clear articulation of vowels in words is related to lower pitch values.

**TABLE 1:** Pitch values, pitch variation, and the articulation index (VAI) of stressed vowels in the words of children from three age groups.

Group of children (years)	Voice characteristics				Articulation	
	F0 (Hz)		F0 (maximum−minimum) (Hz)		VAI (conventional units)	
	Mean ± SD	Median	Mean ± SD	Median	Mean ± SD	Median
4–6	296.5 ± 44.9	301	57.7 ± 56.6	45	0.97 ± 0.17	0.95
6.5–8	292.2 ± 57.6	279	78.2 ± 73.3	45	0.99 ± 0.11	1.02
9–11	254.4 ± 25.9	258	41.5 ± 16.4	43.5	0.94 ± 0.09	0.93

The number of replicas by one word (*p* < 0.05) decreases and by a phrase and two phrases and complex sentences increases with age in children (Table 2).

**TABLE 2:** The structure of response replicas of children from three age groups in the dialogue with the adult (medians).

Group of children (years)	One word	Simple phrase	Two phrases	Complex sentence	Several phrases
4–6	50	38	3.5	1	3.5
6.5–8	44	34	5	4	4
9–11	17	54	7	8	4

With increasing child’s age (from group 1 to 2), VAI (0.408, Spearman correlation *p* < 0.05), the number of replicas represented by a complex sentence (0.532) and replicas represented by several simple sentences (0.611) increase, and the number of replicas by one word (−0.546) decreases. The correlation analysis data are confirmed by regression analysis: the link was found between the child’s age (predictor) and the following indicators: the number of replicas by a complex sentence, *F*(1,23) = 8.774, *p* < 0.007 (β = 0.525, *R*^2^ = 0.276); replicas by several simple sentences *F*(1,23) = 12.119, *p* < 0.002 (β = −0.587, *R*^2^ = 0.345); replicas by one word *F*(1,23) = 11.487, *p* < 0.002 (β = −0.587, *R*^2^ = 0.333).

The correlation between VAI and the reading skills formation (−0.76) is to interpret; the articulation in children reading phrases fluently is not clear because the motor program to pronounce phrases has already been formed, and there is no need to clearly articulate phonemes as in reading syllables and simple words.

The dichotic listening was conducted in 39 children, the determining PFLA – in 24 children. Children with the right-sided preference prevailed in all the groups—the left leading hemisphere by speech (88% children in group 1, 76% children in group 2, 45% children in group 3); the number of children with no preference–ambivalence were 6% in group 1, 8% in group 2, and 11% in group 3.

Profile of functional lateral asymmetry was mainly right-sided in children of all groups: 69%, group 1; 87%, group 2; and 100%, group 3. The ambivalence of PFLA in the younger age group (4–6 years) was revealed in 23% of children and in the middle age group (6.5–8 years) in 13% of children; the left-sided preference was found in only 8% children of the younger age group.

Phonemic hearing in all children was in norm.

Analyzing the EEG pattern, the wide range of individual differences in the alpha, theta, and delta expression in children, particularly of the younger age group, was revealed (Table 3).

**TABLE 3:** The number of children (%) in each age group with the different expression of alpha, theta, and delta in the EEG pattern.

No.	EEG rhythms	The characteristic of rhythm expression	Children in groups (%)		
			4–6	6.5–7	8–11
1	Alpha	Unexpressed	**27**	6	14
	Theta	Expressed			
	Delta	Expressed			
2	Alpha	Unexpressed	3	6	
	Theta	Irregular			
	Delta	Irregular			
3	Alpha	Irregular, unstable	**29**	**19**	
	Theta	Expressed			
	Delta	Expressed			
4	Alpha	Irregular, unstable	9	6	
	Theta	Expressed			
	Delta	Irregular			
5	Alpha	Irregular, unstable	6	6	
	Theta	Irregular			
	Delta	Irregular			
6	Alpha	Irregular, unstable	6	12	**43**
	Theta	Irregular			
	Delta	Expressed			
7	Alpha	Regular, unstable	3	6	**28**
	Theta	Expressed			
	Delta	Expressed			
8	Alpha	Regular, unstable	6	13	
	Theta	Irregular			
	Delta	Irregular			
9	Alpha	**Regular, stable**	6	6	
	Theta	Expressed			
	Delta	Expressed			
10	Alpha	**Regular, stable**	5	**20**	**15**
	Theta	Irregular			
	Delta	Irregular			

*Bold emphasis refers to significant variances in alpha, theta, and delta rhythm expressions in children’s age group.*

The variances in alpha, theta, and delta rhythm expressions in the EEG pattern were represented in a greater number (*n* = 10) in children of the younger and middle age groups than in the older group (*n* = 4). In children of the younger age group, the unexpressed alpha or the irregular unstable alpha in combination with the expressed slow wave activity of theta and delta (27 and 29% of children, respectively) is the most common. In children aged 6.5–8 years, along with the irregular unstable alpha and the expressed theta and delta (19% of children), the regular stable alpha and the irregular theta and delta (19%) were detected. In children of the older age group, in the presence of children with the characteristics of an “adult” EEG, the regular stable alpha and the irregular theta and delta (15% of children), the irregular unstable alpha, irregular theta, and expressed delta prevail (43% of children).

For the sample of children, changes in the generalized alpha rhythm localization in the younger age group to the parieto-occipital area in the older one were revealed. The number of children with generalized alpha rhythm localization in the younger age group was 15%, and in the second group, 25%; children with generalized alpha rhythm localization were absent in the older age group. The number of children with parieto-occipital alpha rhythm localization changed from 85% in the younger age group to 100% in the older one.

Children with no alpha asymmetry prevail in all groups (65%, group 1; 81%, group 2; and 56%, group 3), with the number of children with the rhythm asymmetry (right-sided) in the older age group increasing (26%, group 1; 6%, group 2; and 33%, group 3).

#### Data for the First Group of Children

The statistical data analysis showed that the correlation (Spearman correlation, *p* < 0.05) between the child’s age (4–6 years, preschoolers) and the theta expression in the EEG pattern (0.49), and the alpha expression in the EEG pattern (0.47) was found. The correlation analysis data are confirmed by multiple regression analysis (Table 4): the alpha and theta expressions correlate with the child’s age.

**TABLE 4:** Result of multiple regression analysis: EEG characteristics—intensity of theta and alpha rhythms/child’s age (4–6 years).

Dependent variable: child’s age								

*R*^2^	*F*	Independent variable	Beta	SE of beta	*B*	SE of *B*	*t*	*p* Level
0.283	7.514	Theta rhythm intensity	0.365	0.157	0.65	0.28	2.325	0.027
		Alpha-rhythm intensity	0.333	0.157	0.284	0.134	2.12	0.042

*R^2^, correlation coefficient (R) squared; SE, standard error; B, regression coefficient.*

The alpha localization (centro-parietal region) is negatively related to the frequency of replicas by one word (−0.535, Spearman correlation) [*F*(1,23) = 11.856, *p* < 0.002 (β = −0.583, *R*^2^ = 0.34); regression analysis] and positively correlated by one simple phrase (0.552) [*F*(1,23) = 13.751, *p* < 0.001 (β = 0.612, *R*^2^ = 0.374)] and by a complex sentence (0.511). The alpha asymmetry was negative correlated with the number of replicas by simple phrases (−0.431, Spearman correlation) [*F*(1,23) = 4.685, *p* < 0.041 (β = −0.411, *R*^2^ = 0.169); regression analysis] and positively correlated to replicas by several phrases (0.464).

The theta in the EEG pattern correlates with the number of replicas by a complex sentence (0.4, Spearman correlation), by several phrases (0.46) [*F*(1,23) = 8.352, *p* < 0.008 (β = 0.516, *R*^2^ = 0.266); regression analysis]. The delta expression in the EEG pattern is associated with the pitch values (0.415).

#### Data for the Second Group of Children

For the age group of 6.5–8 years (the first to second grades in the elementary school), the correlation (Spearman, *p* < 0.05) between the theta expression in the EEG pattern and the number of replicas by several simple phrases (0.548) was revealed. The theta expression in the EEG pattern is the predictor of the pitch values (0.667, Spearman correlation), [*F*(1,12) = 10.276, *p* < 0.008 (β = 0.679, *R*^2^ = 0.461); regression analysis], the pitch variation values (0.539); the delta expression correlates with the pitch values (0.664) [*F*(1,12) = 10.768, *p* < 0.006 (β = 0.688, *R*^2^ = 0.473)].

Physiological indices at child’s birth are associated with the characteristics of EEG rhythms: the gestational age correlates with the alpha expression (0.662, Spearman correlation); the Apgar-1 scores, with the theta expression (−0.602); the Apgar-2 scores, with the theta expression (−0.66) [*F*(1,14) = 6.562, *p* < 0.02 (β = −0.565, *R*^2^ = 0.319)].

#### Data for the Third Group of Children

For the age group of 9–11 years (the third to fifth grades in the school), it was established that the number of replicas by one word increases with the child’s age (0.926, the Spearmen correlation) [*F*(1,6) = 6.796, *p* < 0.04 (β = 0.729, *R*^2^ = 0.531); regression analysis].

The alpha asymmetry is related to the number of replicas by one word (−0.78), *F*(1,6) = 9.511, *p* < 0.02 (β = −0.783, *R*^2^ = 0.613). The Apgar-1 scores are associated with the alpha expression (0.774).

#### Spectral Analysis

Based on the spectral analysis of EEG recordings, it was found that with the child’s age, the alpha frequency increases in the left occipital region—lead O1 (0.588, *p* < 0.05; Spearman correlation between the child’s age and the alpha frequency), *F*(1,57) = 21.075, *p* < 0.00002 (β = 0.52, *R*^2^ = 0.27); regression analysis; in the right occipital region—lead O2 (0.526), *F*(1,57) = 15.992, *p* < 0.0002 (β = 0.47, *R*^2^ = 0.219); in the right frontal region—lead F4 (0.26), *F*(1,57) = 5.279, *p* < 0.03 (β = 0.291, *R*^2^ = 0.848).

With the child’s age, the alpha index increases in occipital region—lead O1 (0.397, Spearman correlation), *F*(1,57) = 13.417, *p* < 0.0006 (β = 0.437, *R*^2^ = 0.19); lead O2 (0.412), *F*(1,57) = 12.264, *p* < 0.0009 (β = 0.421, *R*^2^ = 0.177); and in the frontal areas of the cortex—lead F3 (0.382), *F*(1,57) = 8.496, *p* < 0.005 (β = 0.36, *R*^2^ = 0.13); lead F4 (0.346), *F*(1,57) = 5.065, *p* < 0.03 (β = 0.286, *R*^2^ = 0.082).

The child’s birth physiological indicators are associated with the alpha indices; thus, the child’s birth weight correlates with the alpha index at an older age: lead O2 (0.321), *F*(1,40) = 8.912, *p* < 0.005 (β = 0.427, *R*^2^ = 0.182); lead F3 (0.404), *F*(1,40) = 12.025, *p* < 0.001 (β = 0.481, *R*^2^ = 0.231); lead F4 (0.489), *F*(1,40) = 17.677, *p* < 0.0001 (β = 0.554, *R*^2^ = 0.306); the Apgar-1 scores correlated with the alpha index: lead F3 (0.34), *F*(1,47) = 5.241, *p* < 0.03 (β = 0.317, *R*^2^ = 0.1); lead F4 (0.409), *F*(1,47) = 7.326, *p* < 0.009 (β = 0.367, *R*^2^ = 0.135); the Apgar-2 scores correlated with the alpha index in lead F4 (0.27).

The complexity of response replicas of the child in the dialogue is associated with the alpha indices. The number of replicas by one word correlates negatively with the alpha index in the occipital region: leads O1 (−0.416, Spearman correlation), *F*(1,45) = 10.795, *p* < 0.002 (β = −0.44, *R*^2^ = 0.193), regression analysis, and O2 (−0.491), *F*(1,45) = 15.642, *p* < 0.0003 (β = −0.508, *R*^2^ = 0.058).

The quantity of two-phrase sentences (dependent variable) correlates positively with the alpha index in the occipital region: leads O1 (0.31) and O2 (0.39), *F*(1,45) = 4.458, *p* < 0.04 (β = 0.3, *R*^2^ = 0.09). The number of replicas by complex sentences is positively related to the alpha index in the occipital region: leads O1 (0.504), *F*(1,45) = 15.720, *p* < 0.0003 (β = 0.509, *R*^2^ = 0.259) and O2 (0.586), *F*(1,45) = 24.329, *p* < 0.00001 (β = 0.592, *R*^2^ = 0.351); in the frontal region: leads F3 (0.372), *F*(1,45) = 8.547, *p* < 0.005 (β = 0.4, *R*^2^ = 0.16) and F4 (0.39), *F*(1,45) = 9.809, *p* < 0.003 (β = 0.423, *R*^2^ = 0.179); with the alpha frequency in leads O1 (0.359) and O2 (0.316), *F*(1,45) = 10.322, *p* < 0.002 (β = 0.432, *R*^2^ = 0.187). The pitch values correlated with the alpha index in the occipital region: leads O1 (−0.446), *F*(1,44) = 8.594, *p* < 0.005 (β = −0.404, *R*^2^ = 0.163) and O2 (−0.317), *F*(1,44) = 4.968, *p* < 0.03 (β = −0.319, *R*^2^ = 0.101); with the alpha index in the frontal region: leads F3 (−0.42), *F*(1,44) = 9.358, *p* < 0.004 (β = −0.419, *R*^2^ = 0.175) and F4 (−0.41), *F*(1,44) = 8.351, *p* < 0.006 (β = −0.399, *R*^2^ = 0.16).

The data on the alpha frequency and alpha index values in leads O1, O2, F3, and F4 in the age groups of 4–6 years, 6.5–8 years, and 9–11 years are presented in Table 5.

**TABLE 5:** The alpha frequency and alpha index values in children of three age groups: 4–6, 6.5–8, and 9–11 years.

Group of children (years)	O1		O2		F3		F4	
	Alpha frequency	Alpha index	Alpha frequency	Alpha index	Alpha frequency	Alpha index	Alpha frequency	Alpha index
4–6	8.44 ± 0.85	17.8 ± 17.2	8.53 ± 0.74	19.51 ± 19.42	8.26 ± 0.72	8.73 ± 8.97	8.4 ± 1.02	8.45 ± 9.14
6.5–8	9.16 ± 1.03	19.65 ± 18.17	9.25 ± 0.92	18.5 ± 16.99	8.79 ± 1.64	11.99 ± 11.05	8.73 ± 1.2	11.77 ± 11.4
9–11	9.55 ± 0.74	40.0 ± 17.31	9.44 ± 0.85	40.10 ± 17.09	8.35 ± 0.96	16.93 ± 5.32	9.17 ± 0.97	14.18 ± 7.42

The all data analysis (without dividing children into three age groups) revealed that age at the time of EEG registration is related to the alpha rhythm expression in the EEG (0.78), VAI (0.77), and the use of complex sentences by children (0.55). These correlations mean that the older children are characterized by the alpha rhythm expression in the EEG pattern, the clear articulation, and the use of complex sentences in speech, which indicates a high level of speech formation.

Multiple regression analysis revealed that the use of complex sentences, pitch values, and pitch range are predictors of the child’s age (Table 6).

**TABLE 6:** Result of multiple regression analysis: complex phrases frequency, pitch values, and pitch range/child’s age.

Dependent variable: child’s age								

*R*^2^	*F*	Independent variable	Beta	SE of beta	*B*	SE of *B*	*t*	*p* Level
0.559	5.228	Complex phrases	0.643	0.269	0.204	0.085	2.394	0.023
		Pitch values	−0.443	0.162	−0.019	0.007	−2.738	0.01
		Pitch range	0.473	0.192	0.016	0.006	2.458	0.019

*R^2^, correlation coefficient (R) squared; SE, standard error; B, regression coefficient.*

The correlation between acoustic characteristics of the child’s speech and the EEG parameters was revealed: theta rhythm intensity and pitch values in spontaneous speech, *F*(1,44) = 6.384, *p* < 0.015 (β = 0.356, *R*^2^ = 0.127), regression analysis. The pitch depends on the child’s age, *F*(1,44) = 7.393, *p* < 0.009 (β = −0.379, *R*^2^ = 0.144)—the younger the child is, the higher the pitch values are. The relationship between theta rhythm intensity and the pitch variation [F0maximum−F0minimum] is shown (Table 7). The VAI is associated with the asymmetric alpha localization *F*(1,44) = 5.931, *p* < 0.01 (β = 0.345, *R*^2^ = 0.119).

**TABLE 7:** Result of multiple regression analysis: EEG characteristics—theta rhythm intensity/pitch range.

Dependent variable: pitch range								

*R*^2^	*F*	Independent variable	Beta	SE of beta	*B*	SE of *B*	*t*	*p* Level
0.227	2.354	Theta rhythm intensity	0.424	0.171	50.847	20.517	2.478	0.018

*R^2^, correlation coefficient (R) squared; SE, standard error; B, regression coefficient.*

### Study 2—The Specificity of EEG Pattern in Children at Different Stages of Reading Skills Formation

The formation of reading skills is one of the characteristics of child’s speech and cognitive development. The goal of this study was to reveal correlation between the formation of reading skills and the alpha rhythm characteristics of children aged 4–7 years.

Children (*n* = 19) participating in this study were divided into three groups according to their reading ability: group 1 (*n* = 7 children; age, 4.8 ± 0.8 years; median, 5 years old), children cannot read, they know letters; group 2 (*n* = 7; age, 5.7 ± 0.4 years; median, 5.5 years old), they can read syllables and spell; group 3 (*n* = 5; age, 6.8 ± 1.0 years; median, 7 years old), they can read words and phrases.

For children of group 1, it was shown that the alpha activity is unstable, of relatively low amplitude (up to 50 μV) and low frequency (∼8 Hz). In the EEG pattern, the alpha is irregular and unstable (*n* = 3 children; 4, 5, and 5.5 years), not determined (*n* = 2 children; 4 and 5.5 years), two children (5.5 years and 5 years and 11 months—they know letters) have a relatively mature EEG. All the children have high-amplitude bursts of the delta and theta waves. The spatial asymmetry of the centro-parieto-occipital alpha is unstable.

For children of group 2, the alpha is of high amplitude (up to 100 μV), with frequency of ∼9 Hz. Three children (5.5, 6, and 6.3 years) have an expressed and regular high-amplitude parieto-occipital alpha rhythm. Two children (5.5 and 6.4 years) have expressed irregular high-amplitude alpha rhythm; one child (5.5 years) has an irregular, frequency unstable rhythm; and one more child (6.5 years) has a poorly expressed alpha. The slow wave activity is less expressed than in children of group 1. The tendency to the left-sided dominance of the alpha has been revealed.

In children of group 3, the alpha rhythm has the average amplitude (∼80 μV) and the frequency of 10 Hz. Three children (7 years) have a regular alpha; one (5 years and 11 months) had an irregular, unstable, and low-amplitude rhythm; one (7 years) had a regular low-frequency alpha. The spectral analysis of EEG showed the left-sided predominance of the alpha in the parieto-occipital areas.

To clarify the effect of the age of children on the characteristics of alpha rhythm, the children were grouped by age without regard to the formation of their reading skills: 4, 5, 6, and 7 years old.

**FIGURE 1:**
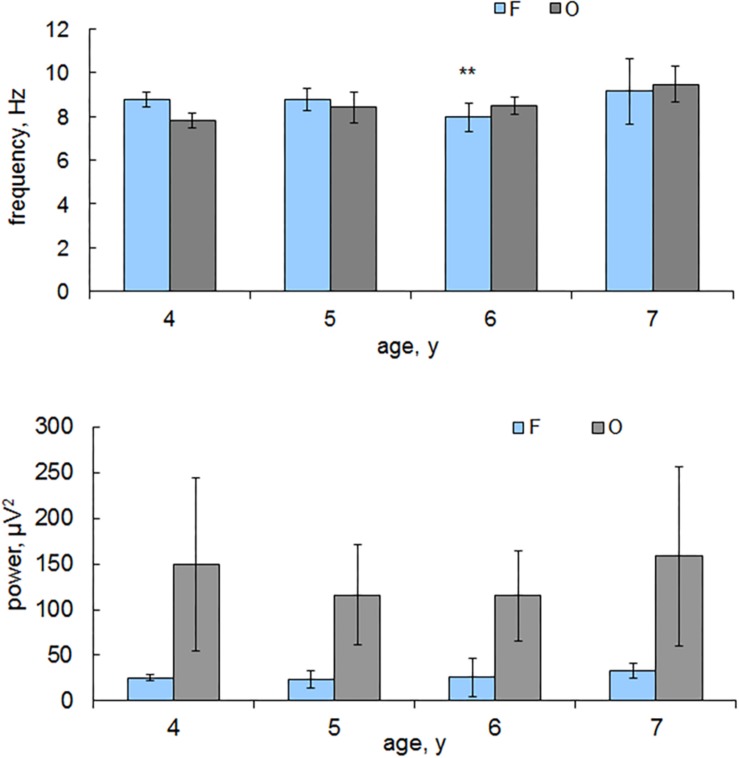
The alpha peak frequency and power in the frontal and occipital areas depending on children’s age. Horizontal axis, the average age of children; vertical axis, the alpha frequency, Hz; power, μV^2^. Blue columns, frontal areas (F); gray columns, occipital (O). Vertical lines are the standard error bars. ^∗∗^indicates the age when the frequency values are minimal, Mann-Whitney test.

The significant (*p* < 0.05) decrease in the alpha frequency in the frontal areas in children from 5 to 6 years old and its increase at the trend level to 7 years were revealed. Significant changes in the alpha frequency values in the occipital areas were not detected (Figure 1).

The ratio of the alpha frequency in the frontal and occipital leads varies depending on the age of the children: from 4 to 5 years, the alpha frequency is higher in frontal areas; from 6 to 7 years, the alpha frequency is higher in the occipital areas. The alpha power in children of all ages is higher in the occipital areas compared with that in the frontal areas (Figure 1). It changes insignificantly in the frontal areas depending on the age of the children and increases in the occipital areas in the children from 6 to 7 years old.

The factor analysis with variables child’s age, the alpha frequency, and power in the frontal and occipital areas, revealed three factors.

Factor 1 is the connection between the alpha power in the frontal (0.88) and occipital (0.93) areas; factor 2 is the connection between the age (0.086) and the alpha frequency in the occipital area (0.85). Factor 3 is an independent parameter—the alpha frequency in the frontal areas (0.98). The direct correlation (*p* < 0.05) is found between the identified factors.

It was revealed that the age dynamics of the alpha is associated with increasing the frequency and power in the frontal and occipital areas, with predominance in the occipital regions.

A correlation between the child’s age (independent variable) and the formation of reading skills (0.763, *p* < 0.05; Spearman correlation) [*F*(1,17) = 23.089, *p* < 0.0002 (β = 0.759, *R*^2^ = 0.576); regression analysis] was found.

The discriminant analysis showed the link between the formation of reading skills and the child’s age [*F*(6,28) = 6.774, *p* < 0.02 (Wilks’ lambda = 0.318, *p* = 0.01)] and the number of replicas by simple phrases (Wilks’ lambda = 0.34, *p* = 0.007).

## Discussion

The results of the study showed the link between characteristics of speech of children such as pitch and its variation, articulation accuracy of vowels in words, complexity of replicas in dialogues with adults, the PFLA, and brain activity in children at the age of 4–11 years. We selected three rhythms—alpha, theta, and delta—for analyzing brain activity, as these rhythms are the most changeable in frontal and posterior regions in the age aspect. Other researchers used the similar approach choosing frontal and posterior regions (Perone et al., 2018b). As pointed out, the frontal region was of particular interest because links between resting-state activity over the frontal region and cognitive development have been observed in previous research (Bell and Fox, 1992; Cuevas et al., 2012).

The originality of our research is to identify the links between various indicators—brain activity and speech features—which characterize the child development. In the study, correlations are shown between characteristics of alpha rhythm in the EEG, VAI, and use of complex sentences by children, i.e., connections reflecting the maturation of brain activity (of alpha rhythm dynamics) and the complication of articulation and grammar. Children aged 9–11 years are characterized by alpha rhythm expression in the EEG pattern, clear articulation, and use of complex sentences in speech that indicates a high level of speech. It was revealed that the alpha rhythm is asymmetrically localized in children with clear pronunciation of words (which determines the intelligibility of their speech) that is typical for children in the middle and older age groups. High values of pitch and its variation in younger children are related to the intensity of theta rhythm in the EEG pattern, as this rhythm is most expressed in younger children.

Along with direct connections, indirect relationships are also shown. The physiological indicators at birth (Apgar score, weight, and gestation age) correlates with the EEG parameters at the age of registration. Considering reading as a certain level of speech formation, the question arises how important the child’s physiological readiness to learn reading is. It is shown that the age at which the child begins to read depends on the level of speech development and the reading learning strategy (Lyakso et al., 2010). The success of the learning correlates with the formation of the child’s reading motivation (Snow, 1993), the child’s individual characteristics in the process of information processing, and the dominant cognitive style.

The data obtained in the EEG analysis of children who are at different levels of reading skills are worthy. The EEG pattern of children at the beginning of mastering reading skills is characterized by “immaturity” that manifests in the low expression of alpha rhythm and in the unclear asymmetry of the alpha. In children reading by syllables, the alpha rhythm is of high amplitude, expressed, with tendency to the left-sided dominance. In children who have mastered reading skills—read words and phrases and understand the meaning of the text—the EEG pattern corresponds to a more “mature” brain. Alpha rhythm is of average and low amplitude, mainly regular, with the left-sided dominance in the parieto-occipital areas. These data indicate the connection between EEG indicators and, consequently, the maturation of brain structures (Huttenlocher and Dabholkar, 1997; Gogtay et al., 2004; Ashtari et al., 2007; Eisermann et al., 2013) and the formation of reading skills, simultaneously with increasing the child’s age, as good-reading children are older. The EEG pattern reflects the level of brain maturation, the measure relatively constant for the child at every age. Our study showed that the alpha rhythm frequency in the occipital and frontal areas in children at the age of 6–7 years is in the range of 8–9 Hz. It is consistent with the data of other researchers (Farber et al., 2000; Bezrukikh et al., 2009).

In general, the study demonstrated the direct and indirect correlations between different indicators, such as brain activity, speech characteristics, and reading skills, in children.

Our further studies will be aimed at studying the EEG pattern in children with ASD of different severity and comparing data on typically developing children.

## Data Availability Statement

The datasets generated for this study will not be made publicly available, the collection of the dataset is still ongoing under the funding of the Russian Science Foundation, and it has not yet passed the licensing process.

## Ethics Statement

The studies involving human participants were reviewed and approved by the Health and Human Research Ethics Committee (HHS, IRB 00003875, Saint Petersburg State University). Written informed consent to participate in this study was provided by the participants’ legal guardian/next of kin.

## Author Contributions

EL: conceptualization and project administration. EL and OF: methodology and resources. OF: validation. EL, OF, and YM: writing—original draft preparation, and writing—review and editing.

## Conflict of Interest

The authors declare that the research was conducted in the absence of any commercial or financial relationships that could be construed as a potential conflict of interest.
